# Genome-wide characterization and expression of *DELLA* genes in *Cucurbita moschata* reveal their potential roles under development and abiotic stress

**DOI:** 10.3389/fpls.2023.1137126

**Published:** 2023-02-23

**Authors:** Weirong Luo, Zhenxiang Zhao, Hongzhi Chen, Wenhong Ao, Lin Lu, Junjun Liu, Xinzheng Li, Yongdong Sun

**Affiliations:** ^1^ School of Horticulture and Landscape Architecture, Henan Institute of Science and Technology, Xinxiang, China; ^2^ Henan Province Engineering Research Center of Horticultural Plant Resource Utilization and Germplasm Enhancement, Xinxiang, China; ^3^ College of Bioengineering, Xinxiang Institute of Engineering, Xinxiang, China

**Keywords:** *Cucurbita moschata*, *DELLA*, characterization, gene expression, abiotic stress

## Abstract

*DELLA* gene family plays a key role in regulating plant development and responding to stress. Currently, many *DELLA* family members have been identified in plants, however, information on *DELLA* genes in pumpkin (*Cucurbita moschata*) is scarce. In this study, physical and chemical properties, gene structure *cis*-regulatory elements and expression of *CmoDELLA* genes were examined in pumpkin. We found that seven *CmoDELLA* genes were identified in pumpkin, and they were unevenly classified into five chromosomes. CmoDELLA proteins were relatively unstable and their secondary structures were mainly made up α-helix and random coil. All seven CmoDELLA proteins contained typical DELLA domain and GRAS domain, however, motif numbers between CmoDELLA proteins were unevenly distributed, implying the complex evolution and functional diversification of CmoDELLA proteins. *Cis*-regulatory elements analysis revealed that *CmoDELLA* genes might play an essential role in regulating plant growth and development, and response to stress in pumpkin. Transcriptome data in the roots, stems, leaves and fruits demonstrated that *CmoDELLA2*, *CmoDELLA3* and *CmoDELLA7* were related to the stems development, *CmoDELLA1*, *CmoDELLA4*, *CmoDELLA5* and *CmoDELLA6* were associated with the fruits development. Furthermore, we found that *CmoDELLA1* and *CmoDELLA5* were up-regulated under NaCl stress. *CmoDELLA1*, *CmoDELLA2*, *CmoDELLA3*, *CmoDELLA5*, *CmoDELLA6* and *CmoDELLA7* were remarkably induced under waterlogging stress. While, all of the 7 *CmoDELLA* genes showed significantly induced expression under cold stress. The expression patterns under abiotic stress suggested that *CmoDELLA* genes might mediate the stress response of pumpkin to NaCl, waterlogging and cold, however, the functions of different *CmoDELLA* genes varied under different stress. Overall, our study provides valuable information for further research about the potential functions and regulatory networks of *CmoDELLA* genes in pumpkin.

## 1 Introduction

DELLA proteins play an essential role in regulating plant development and stress response, as key negative regulators of GA signaling (Zhou et al., 2020). DELLA protein sequences usually contain two conserved domains: DELLA domain and GRAS domain, which are distributed on the N-terminal region and C-terminal region, respectively. DELLA domain is known as the sensing domain of GA signaling for binding to GID1. And GRAS domain contributes mainly to repress GA responses by interacting with transcription factors (TFs), which is crucial for maintaining the functions of DELLA proteins (Xue et al., 2022). DELLA proteins do not contain the typical DNA-binding domain, however, they can interact with many TFs, such as PIFs, BZR1, EXP2, DREB1B, JAZs, and TCPs, to participate in almost all the processes of plant development and stress response (Thines et al., 2007; Navarro et al., 2008; Li et al., 2012; Li et al., 2016; Sechet et al., 2016; Liang et al., 2019). Identification of DELLA proteins firstly began in *Arabidopsis*, and AtRGL1, AtGAI, AtRGL2, AtRGA and AtRGl3, have been found as the members of the *Arabidopsis* DELLA family (Chen et al., 2013). DELLA proteins were subsequently identified from pear (Liu H. et al., 2016), strawberry (Li W.J. et al., 2018), cassava (Li X.L. et al., 2018), *Camellia sinensis* (Han et al., 2020), litchi (Wang et al., 2020), *Brassica napus* (Sarwar et al., 2021), Chinese cabbage (Guan et al., 2021) and other species.

*DELLA* genes play vital roles in regulating seed germination, hypocotyl elongation, plant height, flowering, fruit quality and stress response (Xue et al., 2022). Some studies have shown that *DELLA* genes can control seed germination by regulating the expression of multiple protein kinase genes (Cao et al., 2006). For instance, ABI3 and ABI5 can interact with DELLA proteins to activate SOMNUS and downstream target genes under high temperature, thereby inhibiting seed germination in *Arabidopsis* (Lim et al., 2013). DELLA proteins interacting with ARF6 and PIFs regulate cell elongation of the hypocotyl, which leading to short hypocotyl in *Arabidopsis* (Feng et al., 2008; Oh et al., 2014; Liu et al., 2018). Ethylene interacts with DELLA proteins to inhibit root growth and maintain apical hook-like structure of *Arabidopsis* (Achard et al., 2003). Furthermore, DELLA proteins interact with TCP to influence plant height through regulation of inflorescence apex growth (Davière et al., 2014), and interact with MONOCULM1 to affect tiller number and plant height of rice (Liao et al., 2019). DELLA proteins can also delay the floral transition by interacting with SPLs under long day conditions (Wang et al., 2009; Wu et al., 2009), and repress flowering by inhibiting *LFY* and *SOC1* genes expression under short day conditions (Achard et al., 2007). In addition, Fruit development can be regulated by DELLA proteins. Researchers have concluded that DELLA proteins are implicated in the fruit initiation by interacting with SlARF7/SlIAA9 (Hu et al., 2018), and silencing of *DELLA* gene results in parthenocarpic fruit in tomato (Martí et al., 2007). According to previous studies, DELLA proteins participate in abiotic stress response and improve the plant survival by regulating reactive oxygen species (ROS) levels during adverse environments (Achard et al., 2008a; Achard et al., 2008b). Study has shown that DELLA protein enhances the salt tolerance of wheat seedling by increasing superoxide dismutase (SOD) activity under salt stress (Wang et al., 2016).

Pumpkin (*Cucurbita moschata*) is an annual vegetable crop, which has edible, medicinal and ornamental values. Pumpkin is widely cultivated across the globe, and the top producer is China, which produced 7.7 million tonnes of pumpkin (Worldmapper, 2021). Pumpkin is also widely used as a grafting rootstock for other cucurbit vegetable crops, including cucumber, watermelon and melon, which promotes plant growth and strengthens biotic and abiotic stress tolerance (Liu S. S. et al., 2016; Li et al., 2017; Zhang et al., 2019). In recent years, during cultivation, extreme weather and unfavorable environment conditions such as inappropriate temperature, drought stress, waterlogging stress and salt stress, seriously limited the growth and development of cucurbit vegetable crops, leading to the decline of yield and quality. Therefore, it is of significant importance to elucidate the stress response mechanism of pumpkin and screen pumpkin resistant rootstock for strengthening the stress resistance of cucurbit vegetable crops. However, there is no report of *DELLA* gene family in pumpkin development and stress response.

This main purpose of this study is to identify and characterize *DELLA* gene family in pumpkin, and to uncover their potential functions under abiotic stress. In this current study, seven *CmoDELLA* genes were identified in pumpkin. Moreover, the chromosomal localization, protein properties, phylogenetic analysis, gene structure, promoter *cis*-regulatory elements, protein interaction networks and expression of *CmoDELLA* genes were investigated. Additionally, physiological changes of pumpkin seedlings under waterlogging stress and cold stress were detected. This is the first report on the genome-wide characterization and expression of *CmoDELLA* genes in pumpkin, which provides valuable clues on the biological functions of *CmoDELLA* genes in regulating plant development and stress response for further research.

## 2 Materials and methods

### 2.1 Identification of CmoDELLA family members in pumpkin

Pumpkin protein sequences were downloaded from the cucurbit genomics database (http://cucurbitgenomics.org/). Hidden Markov Model (HMM) profile of the DELLA domain (PF12041) was retrieved from the Pfam database (http://pfam.xfam.org). Then the pumpkin protein sequences were searched for this profile using hmmsearch tool of Tbtools software. *AtDELLA* genes were acquired by searching their gene IDs from TAIR (https://www.arabidopsis.org/), and then they were used as search queries to carry out BLASTp with the E-value of 1e^-5^ against pumpkin protein sequences. Subsequently, all the candidate protein sequences gained with the above two methods were submitted to CDD (http://ncbi.nlm.nih.gov/cdd) in NCBI to reconfirm the CmoDELLA proteins.

### 2.2 Chromosomal localization, physical and chemical properties of *CmoDELLA* genes

The chromosomal localization information of *CmoDELLA* family genes was obtained through cucurbit genomics database, and mapped using TBtools v 1.0986961 software (Chen et al., 2020). According to their chromosomal position, *CmoDELLA* family members were renamed. The physical and chemical properties, such as the coding sequence (CDS) lengths, amino acids number (AA), molecular weight (MW), isoelectric point (pI), grand average of hydropathicity (GRAVY) values and instability index, were predicted through Expasy (https//www.expasy.org/). Additionally, secondary structure prediction was executed *via* NPS@:SOPMA (https://npsa-prabi.Ibcp.fr/cgi-bin/npsa_automat.sopm.html).

### 2.3 Phylogenetic tree, gene structure and protein interaction networks analysis

DELLA protein sequences of pumpkin (7), cucumber (4), melon (4), watermelon (4), *Arabidopsis* (5), soybean (7), *Brassica napus* (13), rice (1), tomato (2) and maize (3) were retrieved from cucurbit genomics database, TAIR and Ensembl database (http://plants.ensembl.org/index.html), respectively. Based on multiple sequence alignment, phylogenetic tree was created using the neighbor-joining (NJ) method with MEGA 7.0. The conserved domains were analyzed by the NCBI CDD, and the conserved motifs were predicted by MEME (https://meme-suite.org/meme/). Furthermore, the distribution maps of conserved domains and conserved motifs were visualized using TBtools v 1.0986961. Promoter sequences (2 kp before the start codon) of *CmoDELLA* genes were analyzed through online PlantCare (https://bioinformatics.psb.ugent.be/webtools/plantcare/html/) to obtain *cis*-regulatory elements. Interaction networks between CmoDELLA proteins and other proteins were conducted through online STRING (https://string-db.org/), using *Arabidopsis* as reference species.

### 2.4 Transcriptome sequencing analysis of *CmoDELLA* genes

The pre-published pumpkin transcriptome sequencing data under four tissues (roots, stems, leaves and fruits) (PRJNA385310) and NaCl stress (PRJNA437579) were obtained from the cucurbit genomics database for exploring the transcriptional profiles. Transcriptional levels were normalized by the reads per kilobase of exon per million reads mapped (RPKM) method. Expression heatmap of *CmoDELLA* genes was generated with TBtools v1.0986961.

### 2.5 Quantitative real-time PCR (qRT-PCR) and physiological indicators measurement under abiotic stress

In this current study, pumpkin variety “Hantailang” was used to explore the expression characterization of *CmoDELLA* genes under waterlogging stress and cold stress. “Hantailang” was cultivated and developed in a climate chamber with growth conditions (16 h light/8 h dark, 25°C daytime/16°C night). The pumpkin seedlings of two-leaf stage were exposed to abiotic stress. Waterlogging stress were conducted with water 2 cm above the soil surface. For cold stress, the seedlings were maintained at 15°C daytime/5°C night. The leaves were collected at day 10 after treatment and stored in -80 °C fridge for qRT-PCR analysis and physiological indicators measurement.

Total RNA of pumpkin leaves was extracted using TaKaRa MiniBEST Plant RNA Extraction Kit (Code No. 9769, TaKaRa, Dalian, China) with the instructions provided in the kit. First chain cDNA was synthesized using PrimeScript™ RT Master Mix (Perfect Real Time) Reagent Kit (Code No. RR036A, Takara, Dalian, China). Subsequently, qRT-PCR experiment was operated by TB Green^®^ Premix Ex Taq™ II (Code No. RR820A, TaKaRa, Dalian, China) to display the expression patterns under waterlogging stress and cold stress. The primer sequences of *CmoDELLA* genes and reference gene (*CmoActin*) were listed in **Table S1**. All the experiments were carried out followed by the instructions. Each sample was replicated three times and the relative expression levels of *CmoDELLA* genes were analyzed with the 2^–ΔΔ^ *^C^* ^t^ method.

The contents of proline and Malondialdehyde (MDA), catalase (CAT) activity in the leaves of pumpkin were measured using proline (Code No. BC0290, Solarbio, Beijing, China), MDA (Code No. BC0025, Solarbio, Beijing, China) and CAT (Code No. BC0205, Solarbio, Beijing, China) assay kit respectively, following the manufacturer’s protocol. SOD activity was determined with SOD assay kit (Code No. G0104F, Geruisi, Suzhou, China).

## 3 Results

### 3.1 Identification and protein properties of *CmoDELLA* gene family

**Figure 1:**
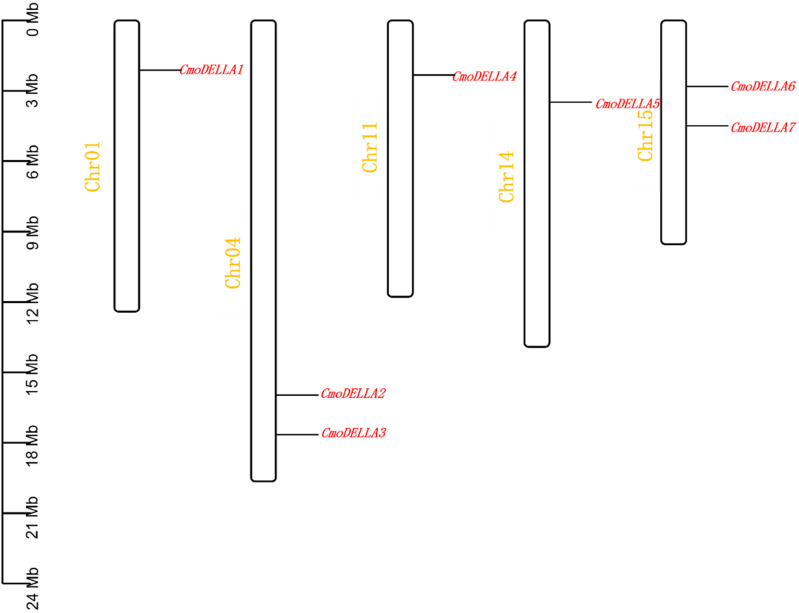
Chromosomal localization of *CmoDELLA* genes. The names of seven *CmoDELLA* genes are shown to the right of each chromosome. Gene position can be measured using the scale on the left of the figure in megabases (Mb).

A total of 7 *CmoDELLA* genes were obtained through genome-wide search, and they were successively renamed as *CmoDELLA1*~*7* respectively, according to their chromosomal localization. Seven *CmoDELLA* genes were located on Chr1, Chr4, Chr11, Chr14 and Chr15, respectively. Among them, there were two *CmoDELLA* genes on the Chr4 (*CmoDELLA2* and *CmoDELLA3*) and Chr15 (*CmoDELLA6* and *CmoDELLA7*), while only one *CmoDELLA* gene on the Chr1, Chr11 and Chr14 (**Figure 1**). The protein properties of *CmoDELLA* gene family were collected in **Table S2**. The CDS lengths of *CmoDELLA* genes varied from 1605 bp to 1854 bp. AA number of CmoDELLA proteins ranged from 534 aa to 617 aa, the MW was between 58316.97 Da and 67383.21 Da, the pI range was 4.70 to 5.52, the GRAVY changed from -0.312 to -0.092. Seven CmoDELLA proteins were defined as unstable proteins with their instability index varying from 42.56 to 53.57. The secondary structures of seven CmoDELLA proteins were all made up α-helix, random coil, extended strand and β-sheets, in which α-helix accounted for the largest proportion (45.52%~49.17%), followed by random coil, extended strand and β-sheets, indicating that the structures of CmoDELLA proteins were the mixed type. Function annotation showed that *CmoDELLA1* and *CmoDELLA5* were related to the regulation of transcription, whereas *CmoDELLA2*, *CmoDELLA3*, *CmoDELLA4*, *CmoDELLA6* and *CmoDELLA7* were involved in the regulation of transcription, plant hormone signal transduction pathway, growth and development, and stress response, indicating their pivotal roles in regulating pumpkin development and stress response.

### 3.2 Phylogenetic tree, sequence alignment and structure of DELLA proteins

**Figure 2:**
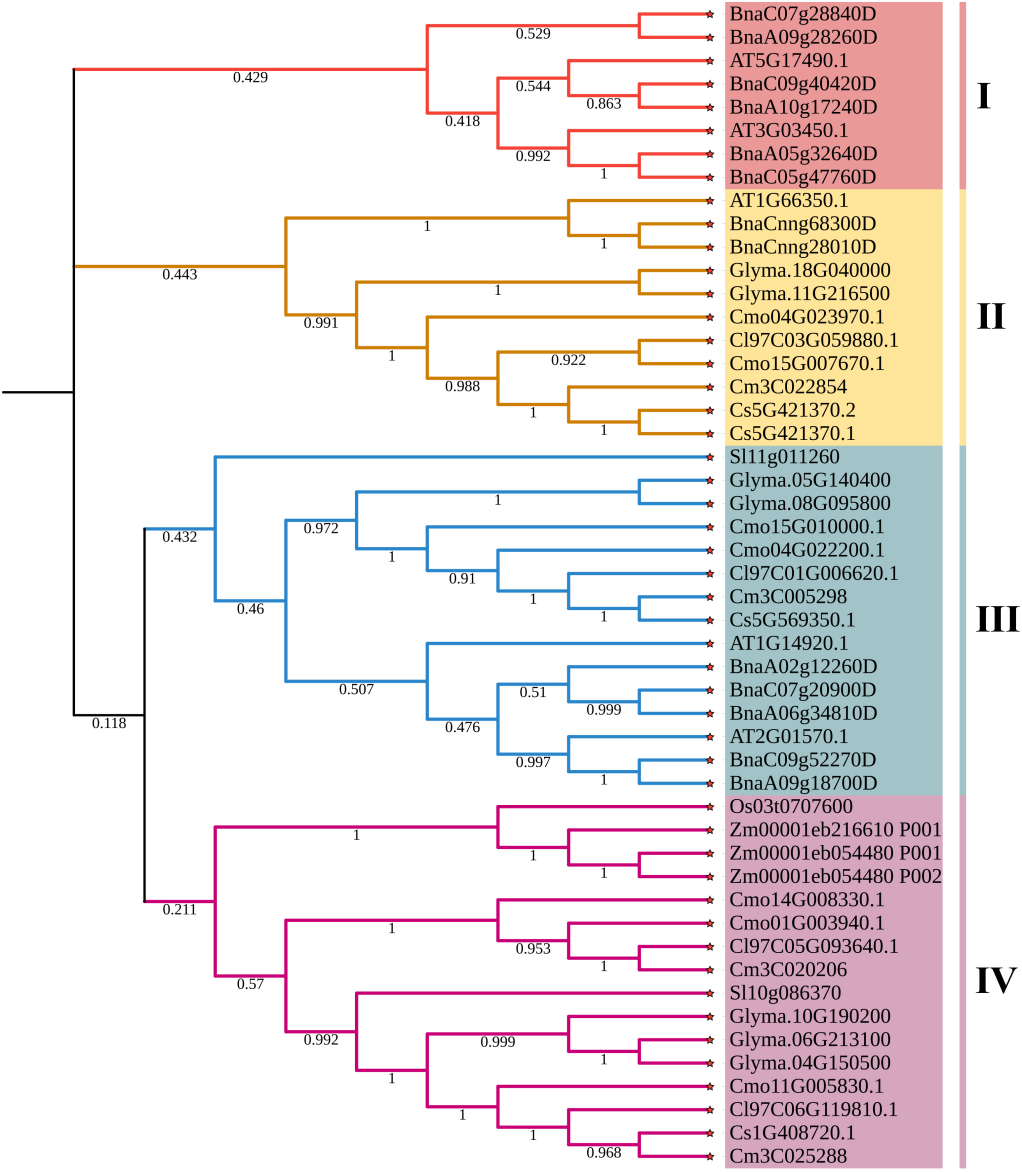
The phylogenetic tree of DELLA proteins from pumpkin, cucumber, melon, watermelon, *Arabidopsis*, soybean, *Brassica napus*, rice, tomato and maize. Cmo: pumpkin, Cs: cucumber, Cm: melon, Cl: watermelon, AT: *Arabidopsis*, Glyma.: soybean, Bna: *Brassica napus*, Os: rice, Sl: tomato, Zm: maize. The 4 major classes are represented by the different colors.

**Figure 3:**
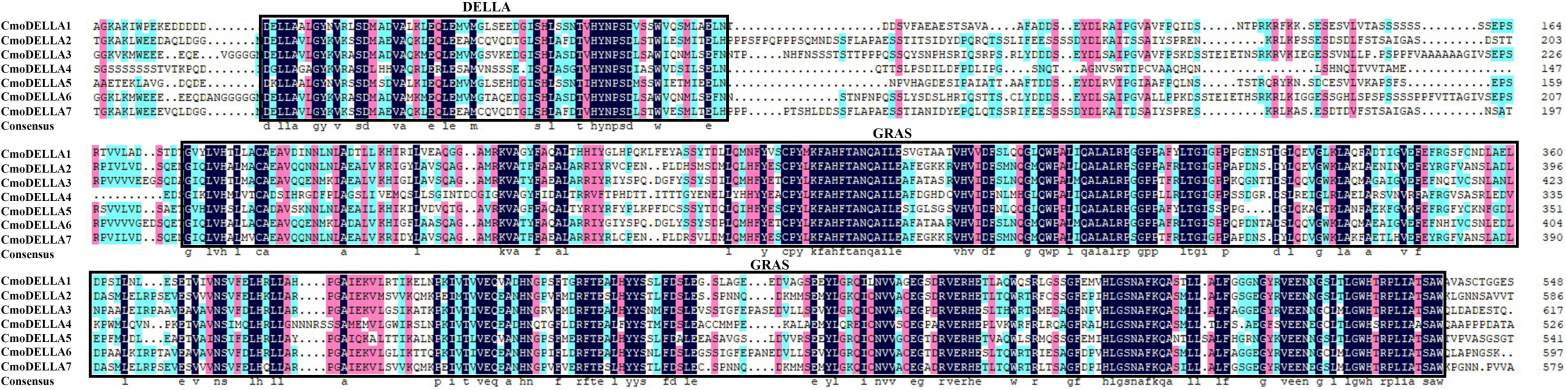
Amino acid sequence alignment of CmoDELLA proteins. The numbers on the right side of the sequence indicate the position of amino acid residues and the colors represent similarities in the protein sequences. The identical amino acid residues are shaded in black background, and the similar amino acid residues are shaded in the red and blue background.

**Figure 4:**
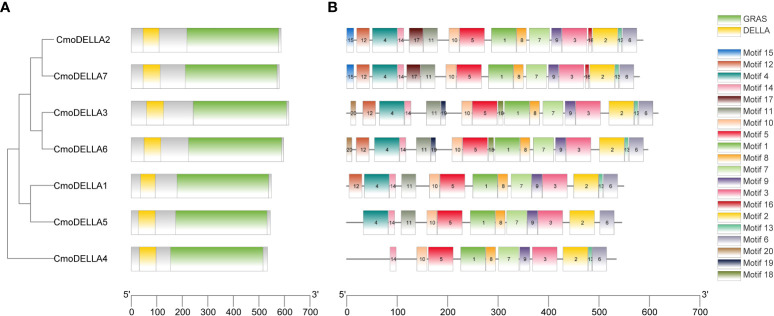
The conserved domains and conserved motifs of CmoDELLA proteins. The lengths of the conserved domains and conserved motifs are shown proportionally. **(A)** The conserved domains of CmoDELLA proteins. DELLA domain and GRAS domain are represented by yellow and green box, respectively. **(B)** The conserved motifs of CmoDELLA proteins. The different conserved motifs are indicated by different colored boxes.

To evaluate the evolutionary relationship of DELLA proteins, phylogenetic tree was created by aligning 50 DELLA protein sequences from pumpkin, cucumber, melon, watermelon, *Arabidopsis*, soybean, *Brassica napus*, rice, tomato and maize (**Figure 2**). According to the phylogenetic relationship listed in the tree, the 50 DELLA proteins could be divided into 4 major classes: Class I, Class II, Class III and Class IV, which contained 8, 11, 15, and 16 members, respectively. As shown in the phylogenetic tree, DELLA proteins from pumpkin exhibited the relatively closer evolutionary relationship with those from watermeolon, melon, cucumber. In addition, CmoDELLA3 and CmoDELLA6 were classified into the Class II, CmoDELLA2 and CmoDELLA7 were clustered into Class III, CmoDELLA1, CmoDELLA4 and CmoDELLA5 belonged to Class IV. Multiple sequence alignment was performed among CmoDELLA proteins (**Figure 3**). The homology between CmoDELLA2 and CmoDELLA7 was the highest at 94.72%, followed by that between CmoDELLA3 and CmoDELLA6 at 89.37%. The homology of other CmoDELLA proteins was more than 64.66%, indicating the high conservation and complex evolution of CmoDELLA proteins. To better understand the structural differences of CmoDELLA proteins, the conserved domains and conserved motifs were detected. The results showed that CmoDELLA proteins were all composed of N-terminal DELLA domain and C-terminal GRAS domain (**Figures 3**, **4A**), but the conserved motifs between CmoDELLA proteins were unevenly distributed (**Figure 4B**, **Table 1**). The number of motifs of different CmoDELLA proteins varied from 11 to 17. Class II memebers contained 17 motifs, Class III memebers included 16 to 17 motifs, whereas Class IV memebers held 11 to 14 motifs. A total of 20 motifs were found in CmoDELLA proteins, and 10 motifs (including motif1, motif2, motif3, motif5, motif6, motif7, motif8, motif9, motif10 and motif14) were highly conserved in all CmoDELLA proteins. Motif4 was identified in all CmoDELLA proteins except CmoDELLA4, and motif13 was present in all CmoDELLA proteins except CmoDELLA5. Moreover, motif15, motif16 and motif17 were detected in CmoDELLA2 and CmoDELLA7. Motif18, motif19 and motif20 were observed in CmoDELLA3 and CmoDELLA6. The different number of motifs between CmoDELLA proteins indicated their functional diversification in pumpkin.

**Table 1:** The motif information in the CmoDELLA sequences.

Motif	Sequence	Number of Amino Acid
motif 1	QMHFYESCPYLKFAHFTANQAILEAFETAARVHVIDFSLNQGMQWPALIQ	50
motif 2	EMYLGRQICNVVACEGSDRVERHETLTQWRTRLESAGFEPIHLGSNAFKQ	50
motif 3	AIEKVLGVIKALKPKIVTVVEQEANHNGPVFMDRFTEALHYYSTLFDSLE	50
motif 4	LGYKVRSSDMADVAQKLEQLEMVMGQVZEDGISHLASDTVHYNPSDLSSW	50
motif 5	SLVHALFACAEAVRVENNNLAEALGKHIRPLIATQAGAMRK	41
motif 6	DSLQEVGWKLAQFAETIGVEFEFRGFVCNNLADLDPSMLELRPEEVEAV	49
motif 7	ALALRPGGPPAFRLTGIGPP	20
motif 8	VATYFAZALARRIYRJYPPKP	21
motif 9	AEYSDDSEYDLKAIPGVAIFPPKDSSTEK	29
motif 10	VFELHRLLARP	11
motif 11	KGZCSSLSGGKAKLWEEEEQEDGGGD	26
motif 12	AGASSEPSRPVVLVDSQETG	20
motif 13	VZSMLSELNNPPS	13
motif 14	EGFRVEENEGCLMLGWHSRPLIAASAWK	28
motif 15	PNNQDKMM	8
motif 16	DSSFLAPAESSTIANIDYEPQRQTSSRI	28
motif 17	MKMKRE	6
motif 18	ETNSRKRLKI	10
motif 19	FECASSYTD	9
motif 20	PQSSQYSDPHHRIQ	14

### 3.3 *Cis*-regulatory elements of *CmoDELLA* genes

**Figure 5:**
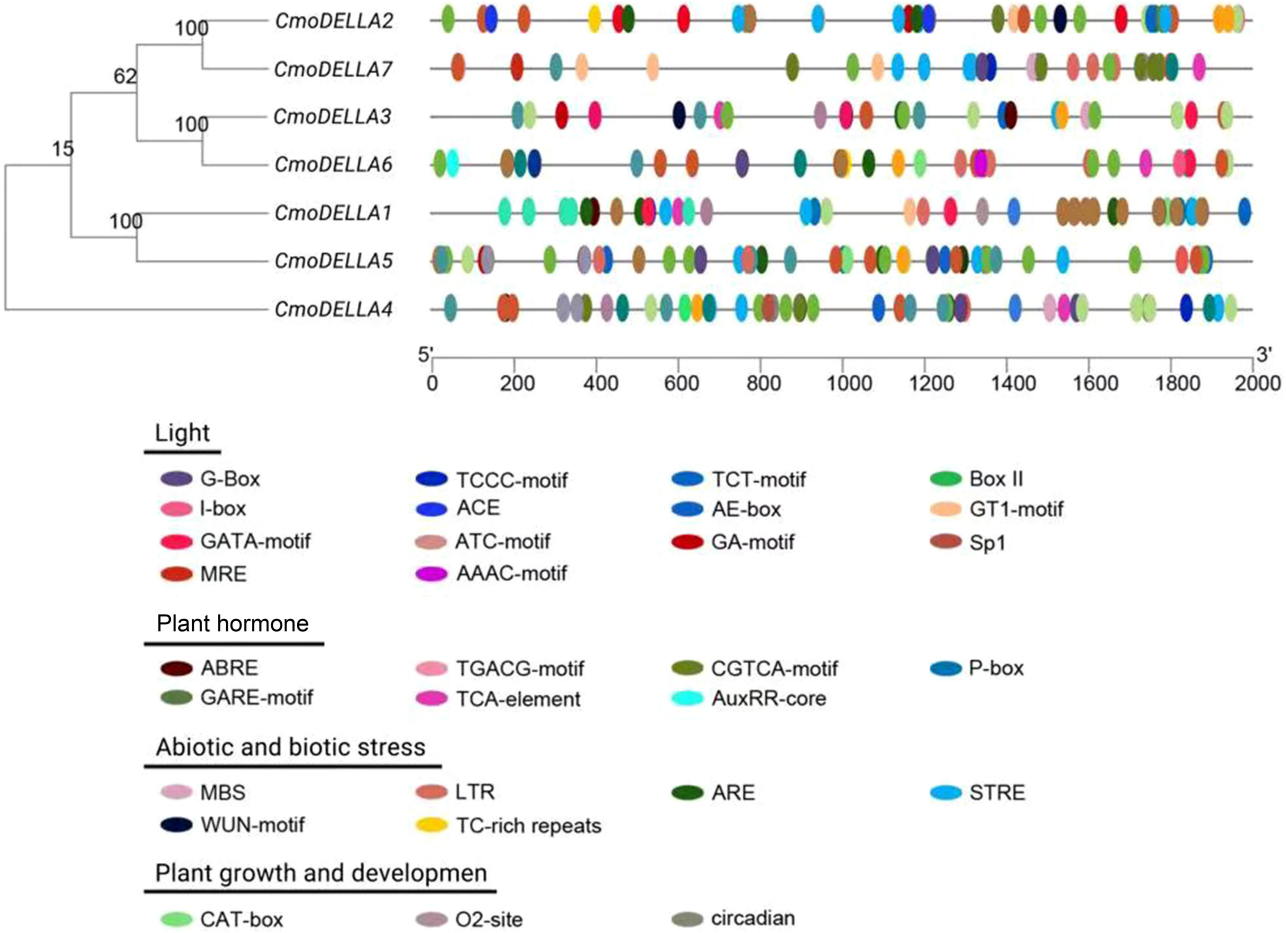
*Cis*-regulatory elements in the promoter regions of *CmoDELLA* genes. These *cis*-regulatory elements are related to light response, plant hormone response, stress response, and plant growth and development. The different colored circles represent the different types and positions of *cis*-regulatory elements in each *CmoDELLA* gene.

To learn more about the potential functions of *CmoDELLA* genes involved in different biological process, the promoter sequences of *CmoDELLA* genes were analyzed to identify *cis*-regulatory elements. The identified *cis*-regulatory elements were mainly related to light, plant hormone, stress, and plant growth and development (**Figure 5**). Among them, light response elements were the most abundant, such as G-Box, TCCC-motif, TCT-motif, Box II, I-box and AE-box. The plant hormone response elements were widely present in the promoter region, including abscisic acid response element (ABRE), TGACG-motif and CGTCA-motif for methyl jasmonic acid response element (MeJA), P-box and GARE-motif for gibberellin response element (GARE), TCA-element for salicylic acid response element (SARE) and AuxRR-core for auxin response element. Stress response elements containing MBS (involved in drought induction), LTR (involved in low temperature response), ARE (involved in anaerobic induction), STRE (involved in heat induction), WUN-motif (involved in wound response), and TC-rich repeats (involved in defense and stress response) were also identified. Simultaneously, we found some growth and development elements, for example, CAT-box associated with meristem, O2-site involved in the regulation of zein metabolism and circadian involved in circadian rhythm regulation.

### 3.4 Interaction networks of CmoDELLA proteins

**Figure 6:**
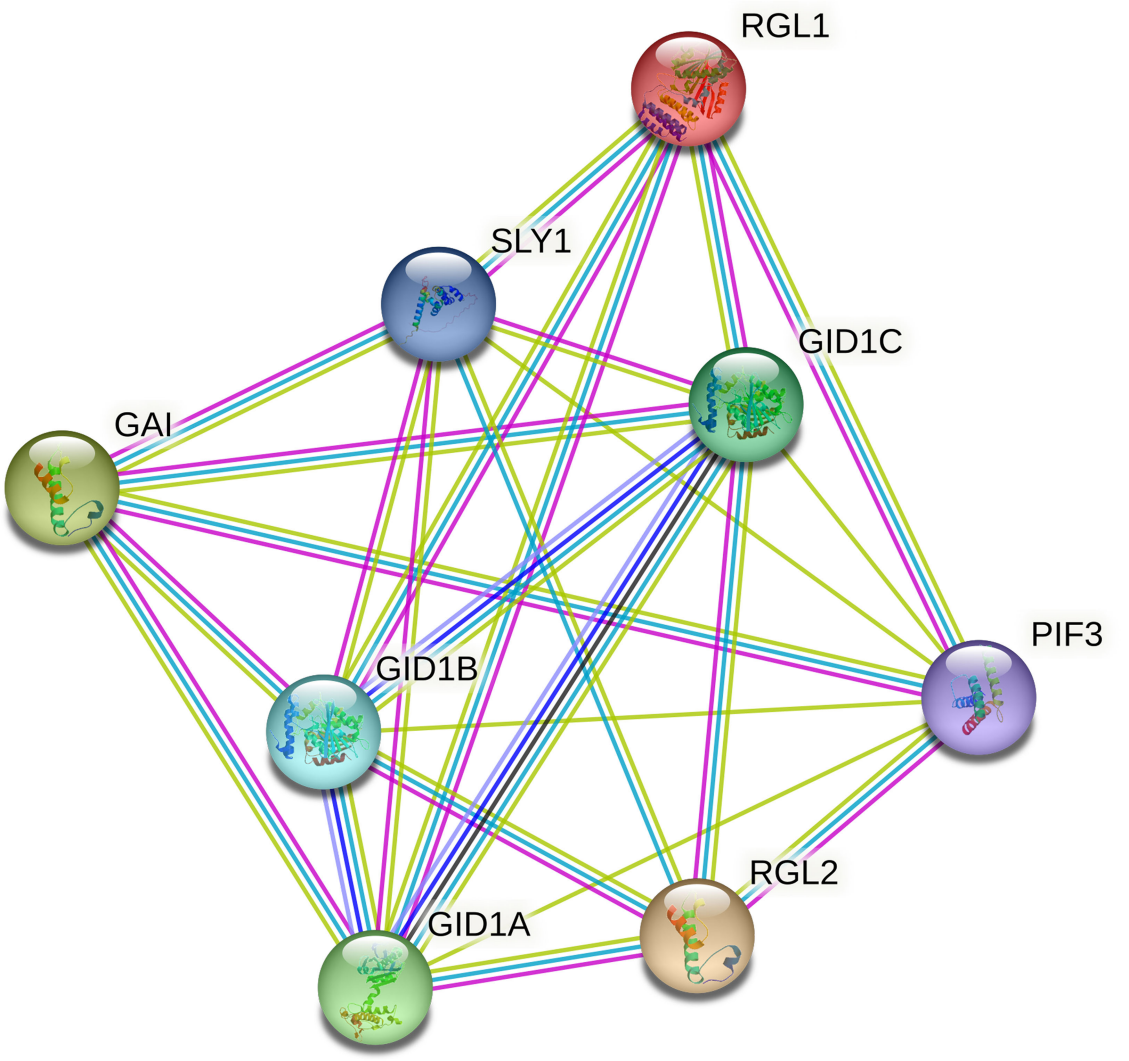
Interaction networks of CmoDELLA proteins. The network nodes represent proteins and the edges indicate protein-protein associations.

To better understand the regulatory mechanism of CmoDELLA proteins, protein interaction networks were constructed. It could be seen from **Figure 6** that seven CmoDELLA proteins, such as RGL1 (CmoDELLA3), GAI (CmoDELLA2, CmoDELLA4, CmoDELLA6 and CmoDELLA7), RGL2 (CmoDELLA1 and CmoDELLA5), all interacted with GID1A, GID1B, GID1C, SLY1 and PIF3 using *Arabidopsis* as reference species.

### 3.5 Tissue-specific expression of *CmoDELLA* genes

**Figure 7:**
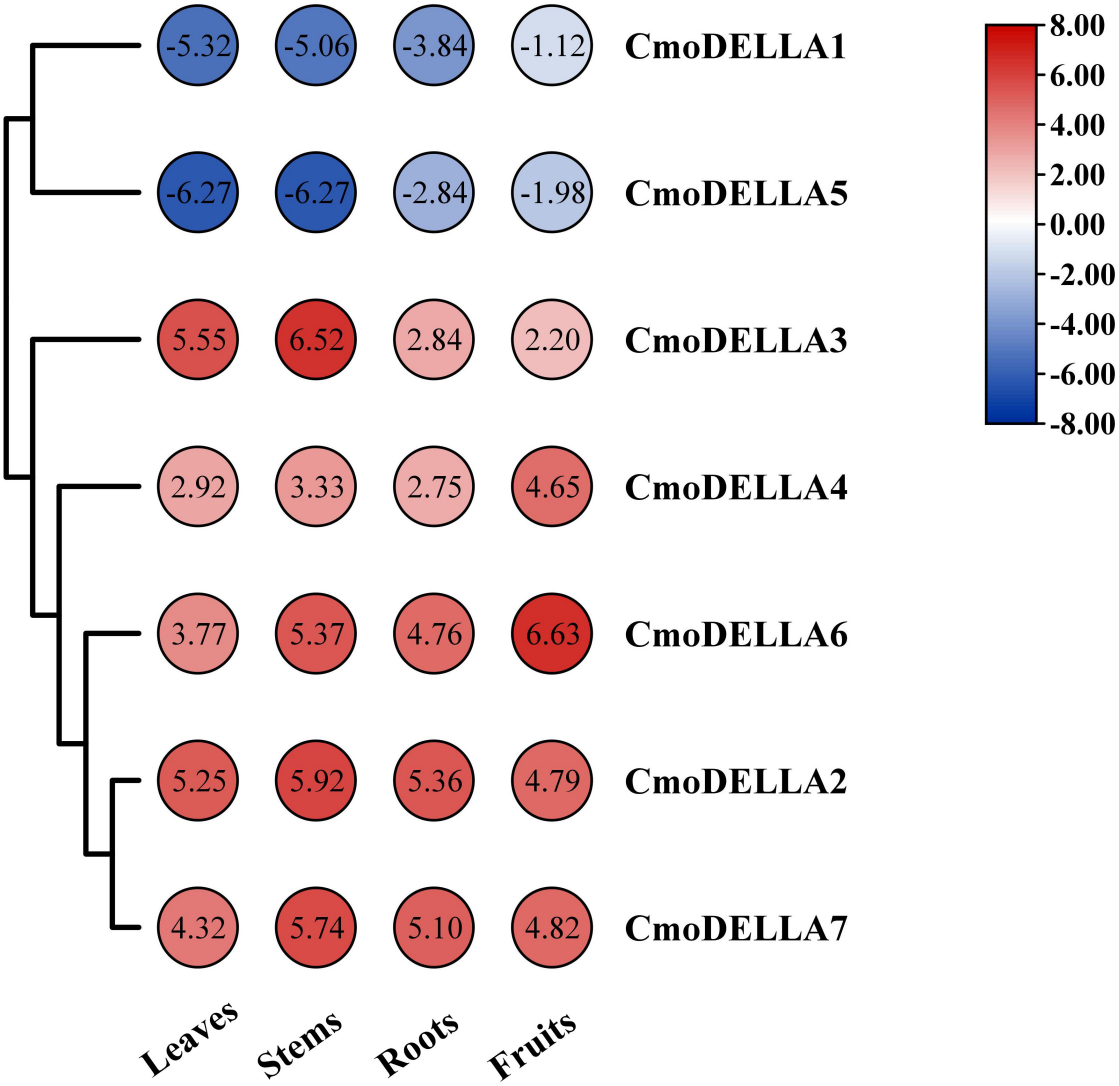
Transcriptional levels of *CmoDELLA* genes in the different tissues (leaves, stems, roots and fruits). The transcriptional levels are normalized by the reads per kilobase of exon per million reads mapped (RPKM) method. The red color represents the higher expression, while the blue color indicates the lower expression.

To uncover the expression profiles of *CmoDELLA* genes, the transcriptional levels of *CmoDELLA* genes in the roots, stems, leaves and fruits were evaluated (**Figure 7**). RNA-seq data revealed that seven *CmoDELLA* genes were expressed in all analyzed tissues but exhibited different abundance levels. For example, *CmoDELLA2*, *CmoDELLA3*, *CmoDELLA4*, *CmoDELLA6* and *CmoDELLA7* had higher transcriptional profiles, whereas *CmoDELLA1* and *CmoDELLA5* demonstrated lower transcriptional levels in the four tissues. Moreover, *CmoDELLA2*, *CmoDELLA3* and *CmoDELLA7* were highly expressed in the stems, while *CmoDELLA1*, *CmoDELLA4*, *CmoDELLA5* and *CmoDELLA6* were mainly expressed in the fruits. Differential expression of *CmoDELLA* genes in different tissues indicated that the tissue specificity and functional divergence of *CmoDELLA* genes in the growth and development of pumpkin.

### 3.6 Transcriptome and qRT-PCR analysis of *CmoDELLA* genes under abiotic stress

**Figure 8:**
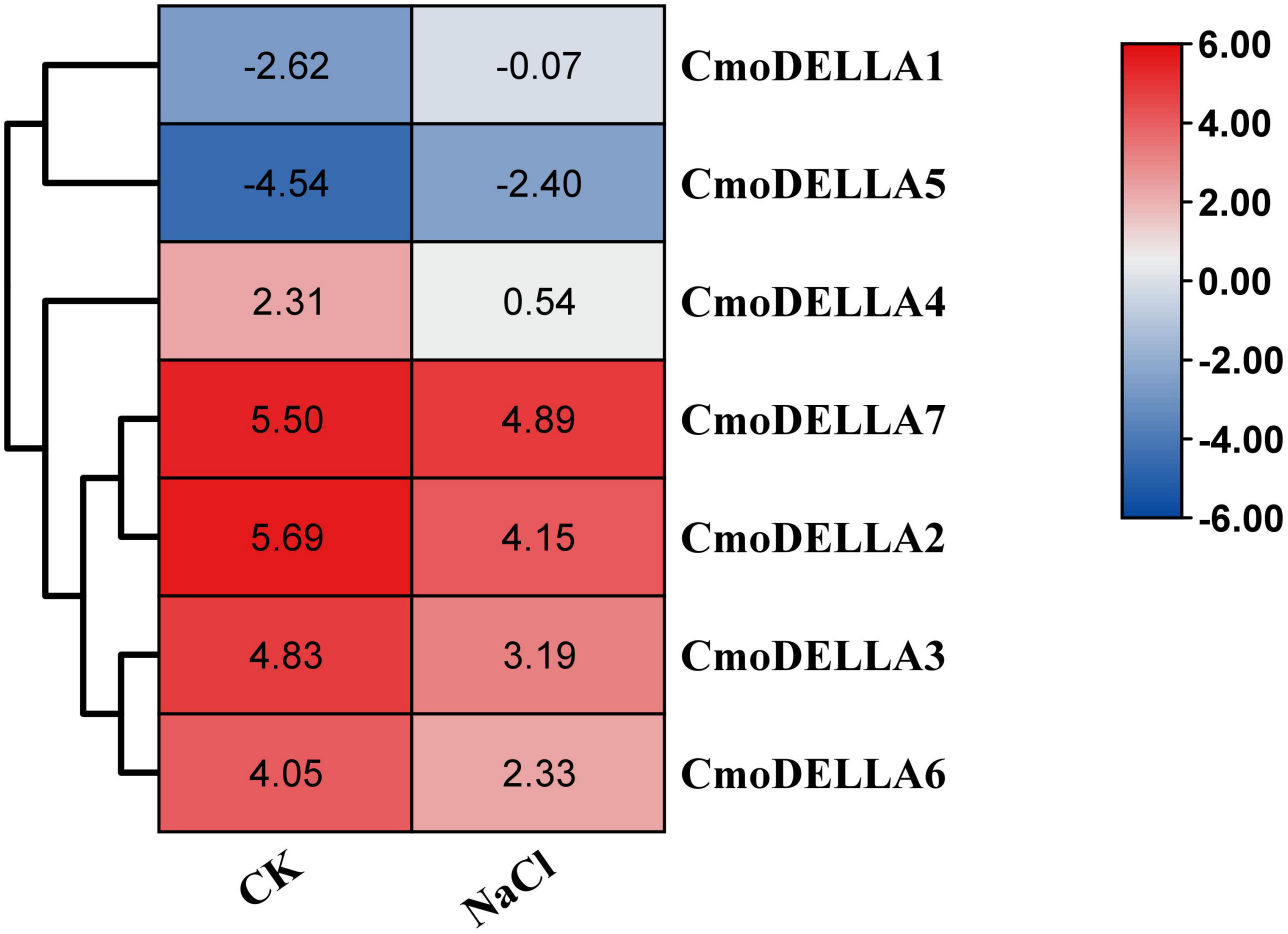
Transcriptional profiles of *CmoDELLA* genes under NaCl stress after 24 h. The transcriptional levels are normalized by RPKM method. The red color represents the higher expression, while the blue color indicates the lower expression.

**Figure 9:**
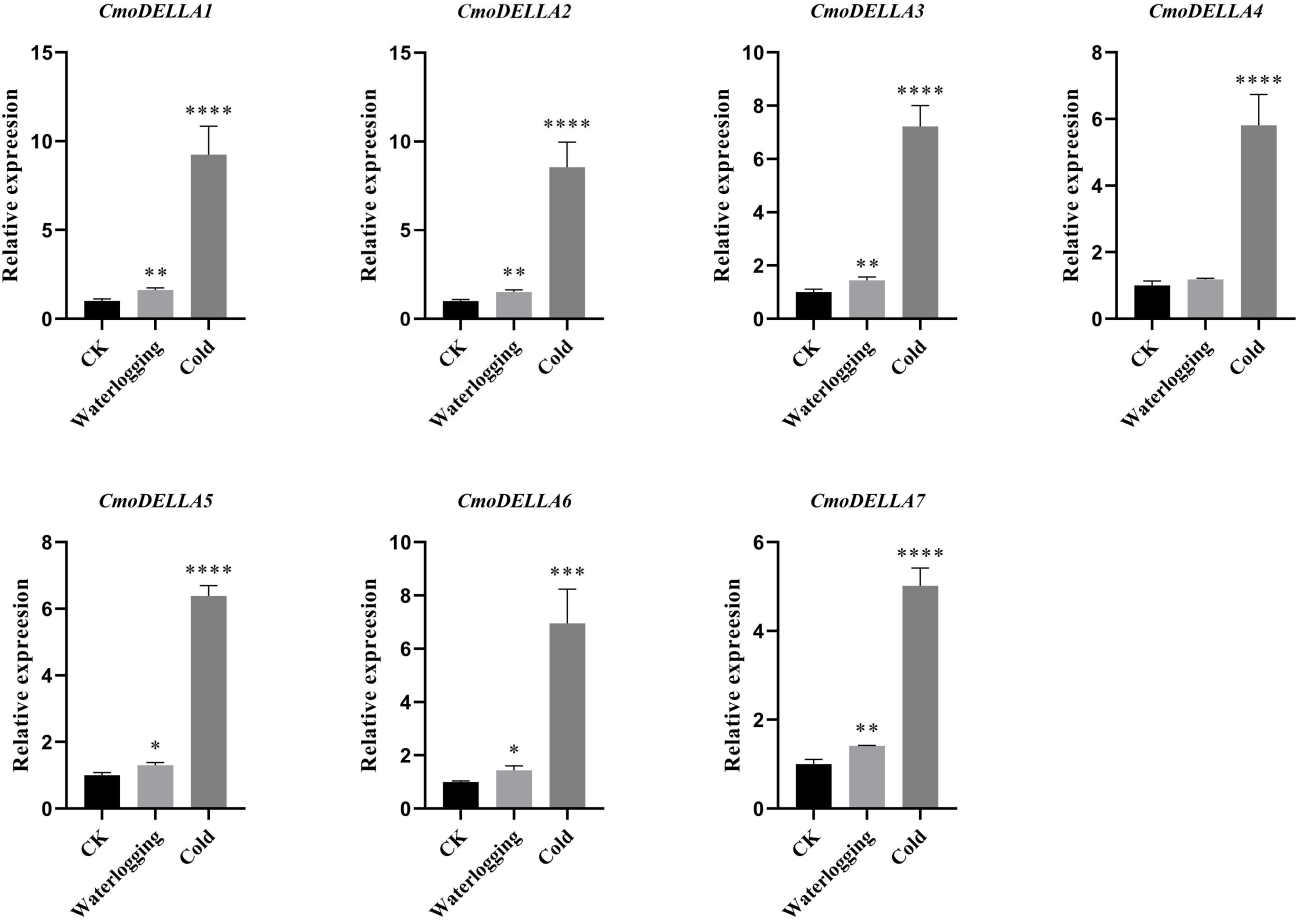
Expression levels of *CmoDELLA* genes in response to waterlogging stress and cold stress after 10 days. The relative expression levels of *CmoDELLA* genes are normalized with respect to the reference gene (*CmoActin*). The X-axis represents the waterlogging stress and cold stress. The Y-axis represents the relative expression levels. The values are denoted as the means ± SDs. The significant difference is represented by asterisks at *P < 0.05, ** *P* < 0.01, *** *P* < 0.001 and **** *P* < 0.0001.

*CmoDELLA* genes not only participated in plant development, but were also involved in various abiotic stress. Promoter *cis*-regulatory elements analysis also showed that *CmoDELLA* genes contained some stress response elements. To further explore and gain more insights into possible functions under abiotic stresses, such as salt, waterlogging and cold temperature, transcriptional profiles of *CmoDELLA* genes under NaCl stress were analyzed based on the pre-published RNA-seq data. *CmoDELLA* genes showed significant differences in response to NaCl stress (**Figure 8**). Transcriptional levels of *CmoDELLA2*, *CmoDELLA3*, *CmoDELLA4*, *CmoDELLA6* and *CmoDELLA7* were down-regulated after 75 mmol/L NaCl stress for 24 h in contrast with the normal condition. In contrast, the expression levels of *CmoDELLA1* and *CmoDELLA5* showed an increased trend after NaCl stress. In addition, the expression levels of *CmoDELLA* genes were investigated by qRT-PCR for further understanding the functions in response to waterlogging stress and cold stress. As shown in **Figure 9**, seven *CmoDELLA* genes were all up-regulated under waterlogging stress and cold stress after 10 days, compared to the normal condition. Under waterlogging stress, the expression levels of *CmoDELLA* genes were significantly higher than that without waterlogging treatment, except for *CmoDELLA4*. For example, *CmoDELLA1*~*7* showed 1.62-, 1.51-, 1.45-, 1.18-, 1.30-, 1.44-, 1.41-fold higher expression levels, respectively, under waterlogging stress. Under cold stress, *CmoDELLA1*~*7* exhited 9.25-, 8.56-, 7.22-, 5.81-, 6.38-, 6.95-, 5.02-fold remarkably higher expression, respectively, compared to the control. The above results suggested that *CmoDELLA* genes might be implicated in response to NaCl stress, waterlogging stress and cold stress in pumpkin, however, the functions of different *CmoDELLA* genes varied under different stress.

### 3.7 Physiological changes under waterlogging stress and cold stress

**Figure 10:**
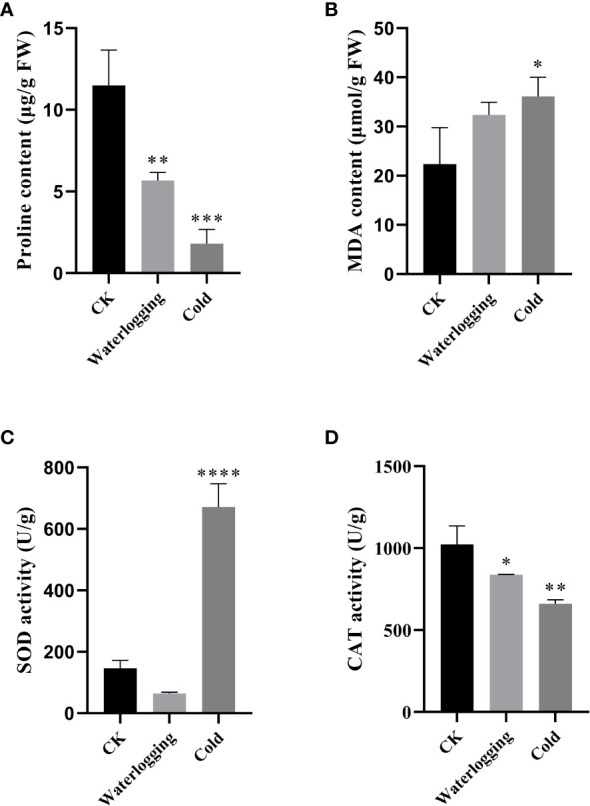
Physiological changes under waterlogging stress and cold stress after 10 days in pumpkin. **(A)**; proline content. **(B)**; MDA content. **(C)**; SOD activity. **(D)**; CAT activity. The values are denoted as the means ± SDs. The significant difference is represented by asterisks at *P < 0.05, ** *P* < 0.01, *** *P* < 0.001 and **** *P* < 0.0001.

In this study, we measured the changes of proline, MDA, SOD and CAT in pumpkin leaves under waterlogging stress and cold stress after 10 days (**Figure 10**). Under waterlogging stress and cold stress, the proline contents were remarkably lower, compared to the control. The MDA contents were 1.44- and 1.62-fold of that in the control, respectively, but did not differ significantly between the control and waterlogging stress. The CAT activities were significantly decreased by 18.16% and 35.43% under waterlogging stress and cold stress, respectively, compared with that in the control. While, SOD activity was lower under waterlogging stress than that in the control, but significantly higher under cold stress than that in the control.

## 4 Discussion

*DELLA* gene family plays key roles in regulating plant development and stress response. Up to now, *DELLA* genes have been extensively identified and characterized in plants, but the research of *DELLA* genes in pumpkin has rarely been reported. In this present study, seven *CmoDELLA* genes were obtained in pumpkin by genome-wide analysis, which were located on five chromosomes respectively. CmoDELLA proteins were all unstable proteins, and the secondary structures of them were mainly made up α-helix and random coil. The phylogenetic tree displayed that 50 DELLA protein sequences from pumpkin, cucumber, melon, watermelon, *Arabidopsis*, soybean, *Brassica napus*, rice, tomato and maize were divided into four subfamilies, and the DELLA proteins of pumpkin shared the closer evolutionary relationship with those of watermeolon, melon, cucumber, which may be due to pumpkin and watermeolon, melon, cucumber belonging to cucurbita family. N-terminal DELLA domain and C-terminal GRAS domain were the typical conserved domains of DELLA family members in various plants (Sarwar et al., 2021). In this study, it was found that seven CmoDELLA proteins held the highly conserved DELLA domain and GRAS domain, shared 10 conserved motifs, however, motif numbers between CmoDELLA proteins were unevenly distributed, indicating the complex evolution and functional diversification of CmoDELLA proteins. These findings were consistent with the study has been found in BnaDELLA proteins (Sarwar et al., 2021).

Promoter *cis*-regulatory elements determine the specific function of the genes. Analysis of *cis*-regulatory elements can provide an insight into exploring the expression and regulation mechanism of genes under different tissues and stress environments. Previous studies have shown that DELLA proteins participate in plant hormone signal transduction pathway, including GA, auxin, abscisic acid, ethylene, and jasmonate (Achard et al., 2006), which affects diverse aspects of plant development and response to environmental stress (Xu et al., 2014). Additionally, some researches have reported the importance of ABRE, SARE and MeJA for abiotic stress tolerance *via* plant hormone signal transduction pathway (Doornbos et al., 2011; Rivas-San Vicente and Plasencia, 2011). In the current study, *CmoDELLA* genes contained a lot of promoter *cis*-regulatory elements involved in light, plant hormone, stress, and plant growth and development, such as ABRE, MeJA, GARE, SARE and ARE, suggesting that the *CmoDELLA* genes may be responsible for plant development and stress response in pumpkin by plant hormone signal pathway. Similar findings have been also reported in apple (Fan et al., 2017), rice (Muhammad et al., 2019), soybean (Liang et al., 2022) and mango (Wang et al., 2022).

Usually, DELLA proteins play essential roles by interacting with other TFs in plants, as key negative regulators of GA signaling (Zhou et al., 2020). GA signal transduction pathway is mainly involved in GID1, DELLA proteins and F-box family proteins (Xue et al., 2022). GID1A, GID1B and GID1C are soluble GA receptors, and SLY1 belongs to F-box family protein. GID1 interacts with the N-terminal domain of DELLA proteins to form GID1-DELLA complex, and then DELLA proteins are bound by SLY1, which leading to degradation of DELLA proteins (McGinnis et al., 2003; Murase et al., 2008). PIF3 is phytochrome-associated protein, and DELLA proteins bind with the promoter of PIF3 to inhibit the PIF-mediated hypocotyl elongation (de Lucas et al., 2008). Protein interaction networks analysis indicated that seven DELLA proteins all interacted with GID1A, GID1B, GID1C, SLY1 and PIF3, suggested their involvement in the development process *via* GA signal transduction pathway.

Gene functions are usually understood by detecting tissue-specific expression profiles of genes. Here, the transcriptome data of *CmoDELLA* genes was analyzed in the roots, stems, leaves and fruits. The results showed that seven genes were all expressed in the four tissues. Moreover, *CmoDELLA2*, *CmoDELLA3* and *CmoDELLA7* were highly expressed in the stems, while *CmoDELLA1*, *CmoDELLA4*, *CmoDELLA5* and *CmoDELLA6* were mainly expressed in the fruits. In cucumber, four *DELLA* genes displayed the distinct expression patterns in the different tissues, and *CsGAIP* exhibited the higher expression levels in the stems (Zhang et al., 2014). In compliance with this, some researches have also been reported that *DELLA* genes have a predominant roles in regulating plant stem elongation growth in *Arabidopsis* (King et al., 2001) and *Brassica napus* (Zhao et al., 2017). Furthermore, we found that *CmoDELLA1* showed significantly up-regulated expression in the pollinated fruits than that in the ovaries without pollination in pumpkin (Luo et al., 2021). Recent study showed that DELLA proteins promoted ovule initiation by interacting with the CUC2 TF (Barro-Trastoy et al., 2022). The results presented here suggested that *CmoDELLA2*, *CmoDELLA3* and *CmoDELLA7* were related to the stems development, *CmoDELLA1*, *CmoDELLA4*, *CmoDELLA5* and *CmoDELLA6* were associated with the fruits development. Further investigation should be conducted to verify their functions.

Several studies have reported that *DELLA* genes participate in the stress response process (Huang et al., 2006). For instance, overaccumulation of DELLA proteins enhances the salt stress (Sakuraba et al., 2017) and cold stress tolerance (Yang et al., 2013), which significantly improves plant fitness. Han et al. (2020) analyzed the transcriptome data of *DELLA* genes in *Camellia sinensis* under drought stress, cold stress and NaCl stress, and speculated that *DELLA* genes were involved in the abiotic stress response. Sarwar et al. (2021) found that *BnaDELLA* genes exhibited different expression abundance under NaCl stress, suggesting the *BnaDELLA* genes vital roles in susceptibility to NaCl stress. Consistent with those, this current study showed the distinct transcriptional patterns of *CmoDELLA* genes under NaCl stress. For instance, *CmoDELLA1* and *CmoDELLA5* showed an increased expression under NaCl stress after 24 h, indicating *CmoDELLA1* and *CmoDELLA5* might be crucial in response to NaCl stress. Additionally, in our qRT-PCR analysis, *CmoDELLA1*, *CmoDELLA2*, *CmoDELLA3*, *CmoDELLA5*, *CmoDELLA6* and *CmoDELLA7* were remarkably induced under waterlogging stress after 10 days. And all of the 7 *CmoDELLA* genes showed significantly induced expression under cold stress after 10 days. These results suggested the vital roles of *CmoDELLA* genes in response to waterlogging stress and cold stress. Many previous studies on *AtDELLA* genes (Zhou et al., 2017; Blanco-Touriñán et al., 2020) and *BnaDELLA* genes (Sarwar et al., 2021) have provided evidence of their fundamental roles in regulating plant physiology under cold stress. Moreover, the expression profiles of different *BnaDELLA* genes varied under different stress treatments (Sarwar et al., 2021). Based on the above results, *CmoDELLA* genes might mediate the stress response of pumpkin to NaCl, waterlogging and cold, however, the functions of different *CmoDELLA* genes varied under different stress.

Previous report has shown that adverse environment conditions promote DELLA accumulation (Achard et al., 2008b) and then DELLA proteins increase SOD activity to improve salt stress tolerance in wheat (Wang et al., 2016). We here showed that *CmoDELLA* genes expression and SOD activity were significantly higher under cold stress than those in the control. Further researches are required to better comprehend the regulatory relationships between *CmoDELLA* genes expression and SOD activity under abiotic stress.

## 5 Conclusions

In summary, seven *CmoDELLA* genes were obtained in pumpkin by genome-wide analysis. Furthermore, the chromosomal localization, protein properties, phylogenetic tree, gene structure, promoter *cis*-regulatory elements and protein interaction networks of *CmoDELLA* genes were conducted. Expression profiles of *CmoDELLA* genes under different tissues and abiotic stress were determined through RNA-seq data and qRT-PCR. Additionally, physiological changes of pumpkin seedlings were measured under waterlogging stress and cold stress. As a whole, these results revealed the vital roles of *CmoDELLA* genes in regulating plant development and stress response in pumpkin, which would provide valuable clues for further studying the potential functions and regulatory networks of *CmoDELLA* genes.

## Data availability statement

Publicly available datasets were analyzed in this study. This data can be found here: NCBI PRJNA385310 and PRJNA437579.

## Author contributions

YS conceived and designed the experiments. WL carried out the experiments. WL, ZZ, HC, WA, LL, and JL analyzed the data, prepared figures and tables. WL wrote the manuscript. YS and XL reviewed the manuscript. All authors contributed to the article and approved the submitted version.
