# Supplementary material for: Genome-wide characterization and expression of DELLA genes in Cucurbita moschata reveal their potential roles under development and abiotic stress
Source: Front Plant Sci. 2023 Feb 23;14:1137126. doi: 10.3389/fpls.2023.1137126 (PMC9995975; doi:10.3389/fpls.2023.1137126)
Supplement: Supplementary file 1 [file Table_1.docx]

Supplementary Material

**Supplementary Table 1: Primer sequences for *CmoDELLA genes***

| Primer | Primer sequence（5ˊ-3΄） |
| --- | --- |
| *CmoActin-F* | GTGCCTGCTATGTATGTTGCC |
| *CmoActin-R* | GGTCCAAACGGAGAATGGCATG |
| *CmoDELLA1-F* | ACCGACACTGGCGTCTATC |
| *CmoDELLA1-R* | TGTGGGTGCAATCCGTAAA |
| *CmoDELLA2-F* | CTCGCAGGAGAACGGAATT |
| *CmoDELLA2-R* | TCAGGGCAGACCCGATAGA |
| *CmoDELLA3-F* | TCACCGAGGCGTTACATTA |
| *CmoDELLA3-R* | CAAGCCACCACATTACAAA |
| *CmoDELLA4-F* | GAAATGGTTCTGGGATGGA |
| *CmoDELLA4-R* | CGAACATGGTGGAGTAGTAAA |
| *CmoDELLA5-F* | TGAGGTCGGAGGAGTATTT |
| *CmoDELLA5-R* | CAGAGTATTCGCCTGCTTG |
| *CmoDELLA6-F* | TGCGCTTCTGCCTCCTAAA |
| *CmoDELLA6-R* | ACAATCCCTGCCGTGGTAA |
| *CmoDELLA7-F* | AAGGTTCTGTCGGTGGTGA |
| *CmoDELLA7-R* | AAACAGCGTCGAGTAGTAATG |
