# Supplementary material for: Genome-wide characterization and expression of DELLA genes in Cucurbita moschata reveal their potential roles under development and abiotic stress
Source: Front Plant Sci. 2023 Feb 23;14:1137126. doi: 10.3389/fpls.2023.1137126 (PMC9995975; doi:10.3389/fpls.2023.1137126)
Supplement: Supplementary file 2 [file Table_2.docx]

Supplementary Material

**Supplementary Table 2: Physical and chemical properties of CmoDELLA proteins**

| Gene Name | Gene ID | CDS (bp) | AA | Mw (Da) | pI | GRAVY | Instability Index | α-helix (%) | random coil (%) | extended strand (%) | β-sheets (%) |
| --- | --- | --- | --- | --- | --- | --- | --- | --- | --- | --- | --- |
| *CmoDELLA1* | CmoCh01G003940.1 | 1650 | 549 | 59429.35 | 4.70 | -0.136 | 42.56 | 47.72 | 38.62 | 8.74 | 4.92 |
| *CmoDELLA2* | CmoCh04G022200.1 | 1764 | 587 | 65092.53 | 5.16 | -0.312 | 51.39 | 47.21 | 33.04 | 12.83 | 6.92 |
| *CmoDELLA3* | CmoCh04G023970.1 | 1854 | 617 | 67383.21 | 5.43 | -0.248 | 52.38 | 45.54 | 38.90 | 9.40 | 6.16 |
| *CmoDELLA4* | CmoCh11G005830.1 | 1605 | 534 | 58316.97 | 5.52 | -0.092 | 43.65 | 47.00 | 39.51 | 8.24 | 5.24 |
| *CmoDELLA5* | CmoCh14G008330.1 | 1638 | 545 | 59685.45 | 5.40 | -0.133 | 45.07 | 49.17 | 36.51 | 9.17 | 5.14 |
| *CmoDELLA6* | CmoCh15G007670.1 | 1794 | 597 | 65022.23 | 4.98 | -0.215 | 53.57 | 46.06 | 41.21 | 8.21 | 8.21 |
| *CmoDELLA7* | CmoCh15G010000.1 | 1743 | 580 | 64537.92 | 5.10 | -0.268 | 47.39 | 45.52 | 40.00 | 8.97 | 5.52 |
